# BRAIN FOG AND A SHRINKING WORLD: GRIEF AND LOSS IN THE CONTEXT OF DEMENTIA

**DOI:** 10.1093/geroni/igae098.1497

**Published:** 2024-12-31

**Authors:** Jen Hirsch, Allison Gibson

**Affiliations:** Michigan State University East Lansing, East Lansing, Michigan, United States; Saint Louis University, St Louis, Missouri, United States

## Abstract

To date, little is known about people living with dementia’s experience of relating to and coping with their disease. Most studies about dementia have focused on their experience from the perspective of a care partner(s), professional care provider(s), or through observation of rather than engaging those living with dementia directly to reflect on their experiences. This qualitative study engaged 20 people living with dementia in focus groups to explore their lived experiences of grief and loss in the context of dementia, such as needing to constantly adapt to changes in disease symptoms, brain fog, and having their environment shrink as they disengage from life roles. Reflexive thematic analysis was used to describe and interpret the rich data. Participants discussed numerous strategies to cope with their diagnosis including engaging in new activities such as hobbies or advocacy efforts around dementia. Others discussed leaning on support systems and peer support groups using social engagement and technology as their most effective coping mechanisms. The themes from this study offer a richer understanding of the experience of dementia as a serious illness. Grief responds well to social support, and yet this population who are dealing with compounding loss also face isolation due to their treatment by others. Understanding this population’s experience and what strategies they use to cope could be used to improve quality of life and psychosocial support for people with dementia and their families.

